# LexiCAL: A calculator for lexical variables

**DOI:** 10.1371/journal.pone.0250891

**Published:** 2021-04-30

**Authors:** Qian Wen Chee, Keng Ji Chow, Winston D. Goh, Melvin J. Yap

**Affiliations:** National University of Singapore, Singapore, Singapore; Nagoya University, JAPAN

## Abstract

While a number of tools have been developed for researchers to compute the lexical characteristics of words, extant resources are limited in their useability and functionality. Specifically, some tools require users to have some prior knowledge of some aspects of the applications, and not all tools allow users to specify their own corpora. Additionally, current tools are also limited in terms of the range of metrics that they can compute. To address these methodological gaps, this article introduces LexiCAL, a fast, simple, and intuitive calculator for lexical variables. Specifically, LexiCAL is a standalone executable that provides options for users to calculate a range of theoretically influential surface, orthographic, phonological, and phonographic metrics for any alphabetic language, using any user-specified input, corpus file, and phonetic system. LexiCAL also comes with a set of well-documented Python scripts for each metric, that can be reproduced and/or modified for other research purposes.

## Introduction

In psycholinguistic research, large databases of words, such as the English Lexicon Project (ELP [1]), the CELEX lexical database [2], the Hoosier Mental Lexicon (HML [3]), the MRC psycholinguistic database [4], and the Auditory English Lexicon Project (AELP [5]), have been essential resources for researchers who require data for a multitude of lexical characteristics. These databases typically consist of a comprehensive set of words and their corresponding lexical properties (e.g., word length, number of syllables, pronunciation, etc.), which allow researchers to generate stimuli selection for experimental design (e.g., selecting words that vary on some dimension while matched on others). Such databases of word properties are nicely complemented by megastudies, in which researchers collect behavioral data (e.g., lexical decision performance) for very large sets of stimuli which are defined by the language rather than by a limited set of criteria [6]. For example, the ELP contains lexical decision and speeded pronunciation performance for over 40,000 monosyllabic and multisyllabic words. The powerful combination of normative databases and megastudies allow researchers to evaluate the influence of various lexical properties on task performance.

However, even though many of these databases are comprehensive and have been made widely available, they are associated with methodological limitations. Most critically, researchers are not able to obtain data for stimuli that are not present in these databases, such as less common words, or stimuli that do not form actual words. For example, in lexical decision paradigms, wherein participants are required to distinguish between actual words and made-up words (e.g., ‘murp’), nonwords are necessary as part of the experimental stimuli. Some researchers are also interested in creating specific stimuli, such as pseudohomophones (e.g., ‘brane’) and illegal nonwords (e.g., ‘btese’), to examine the mechanisms underlying word recognition [7]. These types of stimuli are not typically represented in existing databases, and thus, manipulating and controlling for the lexical characteristics of these stimuli can be difficult without some programming expertise.

### Current methods

In order to overcome the above limitation, a number of tools have been developed to help researchers compute various lexical properties for any stimuli. These tools range from online calculators [8–10] to downloadable programs (e.g., N-watch [11]) and R packages (e.g., ‘LexFindR’ [12]; ‘LexOPS’ [13]; ‘vwr’ [14]), and provide options for researchers to compute specific lexical variables for any user-specified input (both actual words and made-up letter strings).

However, despite the growing number of tools that have been created to aid researchers in obtaining lexical properties for any stimuli, existing tools are still largely limited in terms of their user-friendliness and functionality. First, some tools require users to have some prior knowledge of certain aspects of the respective applications. For instance, using R packages requires basic understanding of the syntax used in R, and would be difficult if users are not familiar with any programming language. Second, some of these tools only recognize certain phonetic systems (i.e., symbols to represent speech sounds in pronunciations) for stimuli input. For example, both the phonotactic probability calculators in [8] and [10] only recognize the computer-readable Klattese phonetic symbols [10]. Third, some of these tools only provide for the calculation of a specific lexical variable, so researchers would still have to find some way to obtain data for other variables of interest.

Furthermore, while some of these tools allow users to specify their own corpora that the calculations are based on (e.g., N-watch [11]; ‘vwr’ [14]), most of them still use built-in corpora from existing databases. For example, the online calculator in [10] computes phonotactic probability with respect to the words in the HML [3], while the online calculator in [9] makes computations based on the child corpora of American English [15,16]. While using fixed corpora is convenient, they may not always be representative of the vocabulary of the study population. Specifically, fixed corpora are meant to approximate the lexicon of a typical participant; for instance, the ELP [1] estimates the size of the adult lexicon to be about 40,481 different words, while [15] estimates the size of the lexicon of kindergarten children to be about 3,728 different words. These estimates may not be applicable to other populations (e.g., children of different ages, elderly subjects, subjects with neuropsychological impairments, etc.), which may be of interest to some researchers [17,18]. Computing lexical properties with respect to these corpora may therefore result in inaccurate estimates, but unfortunately, most of these tools do not allow users to specify the size and contents of the corpora.

## LexiCAL

**Fig 1:**
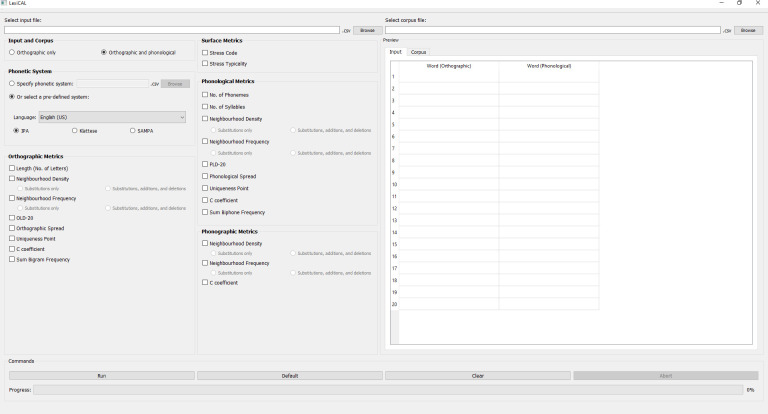
Main window of the application.

To improve on current methods, this article introduces LexiCAL (*lexi*cal *cal*culator), a standalone executable program that serves as a calculator for a wide range of lexical variables. LexiCAL provides options for users to calculate surface, orthographic, phonological, and phonographic metrics, using inbuilt algorithms. These algorithms were also used to compute the metrics in the AELP [5]. The range of metrics that LexiCAL can compute have been shown to be theoretically important predictors of both visual and spoken word recognition [5,19,20]. Importantly, LexiCAL offers users the flexibility of performing calculations for any user-specified input, with reference to any user-specified corpus and phonetic system. Since the algorithms in LexiCAL are generic, LexiCAL allows users to compute metrics for any letter string (both words and nonwords) in any language with an alphabetic script. With a simple and intuitive user interface, LexiCAL provides maximum functionality and useability. Fig 1 shows the main window of LexiCAL.

### Downloading and compatibility

The program (LexiCAL.exe), along with the Python scripts used to compile the executable, can be downloaded as supplementary material with this article. To ensure continued availability of the program, the latest version of LexiCAL is also available at https://osf.io/ydh6e/ and https://inetapps.nus.edu.sg/aelp/other-resources. Each download will come with the license for LexiCAL, and several open-source components are also listed with the license information.

LexiCAL is available only for the 64-bit version of the Windows operating system, and is not compatible with the 32-bit version of Windows, nor the macOS or Linux operating systems. The program is a standalone executable (LexiCAL.exe) that does not require any installation to launch.

### Overview of operations

LexiCAL computes and returns the output for each target word in the user-specified input (the input file), based on the user-specified corpus (the corpus file) and phonetic system.

#### Input file

**Fig 2:**
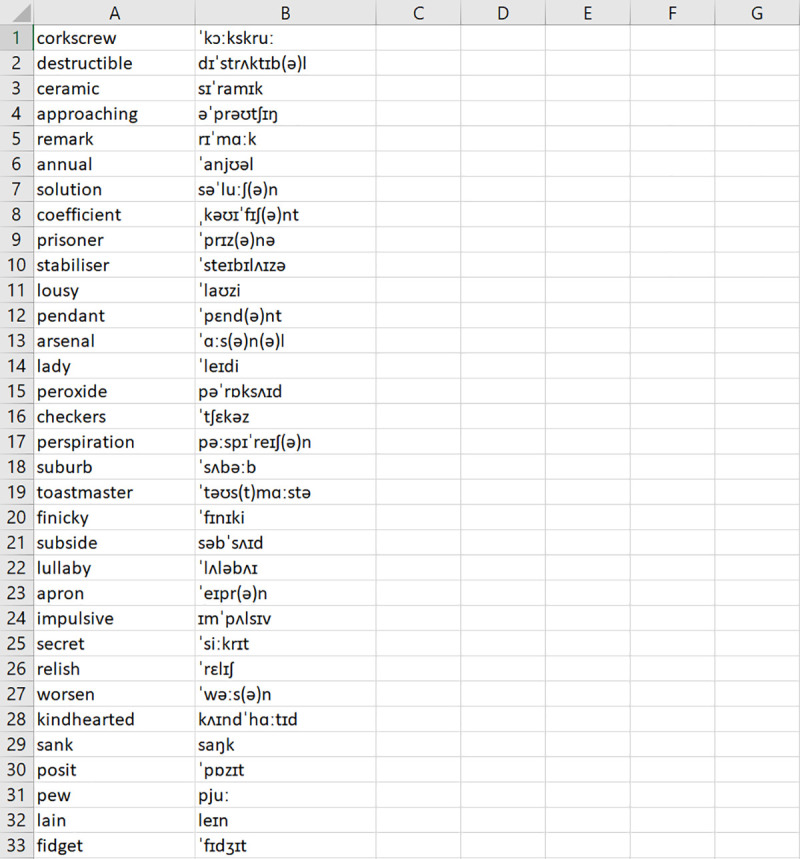
Example of a CSV UTF-8 (comma delimited) input file. Orthographic forms are listed in the first column, and corresponding pronunciations (if applicable) are listed in the second column.

LexiCAL reads target words from a user-specified input file. Users should save target words in a single CSV UTF-8 (comma delimited) file on their machine, without any column headers, and direct LexiCAL to the input file by using the ‘Browse’ option. Orthographic forms (i.e., spelling) should be listed in the first column, and corresponding pronunciations, if applicable, should be listed in the second column. The preview window in LexiCAL will show a preview of the first 20 items in the input file once it has been selected. Fig 2 shows an example of the input file.

#### Corpus file

**Fig 3:**
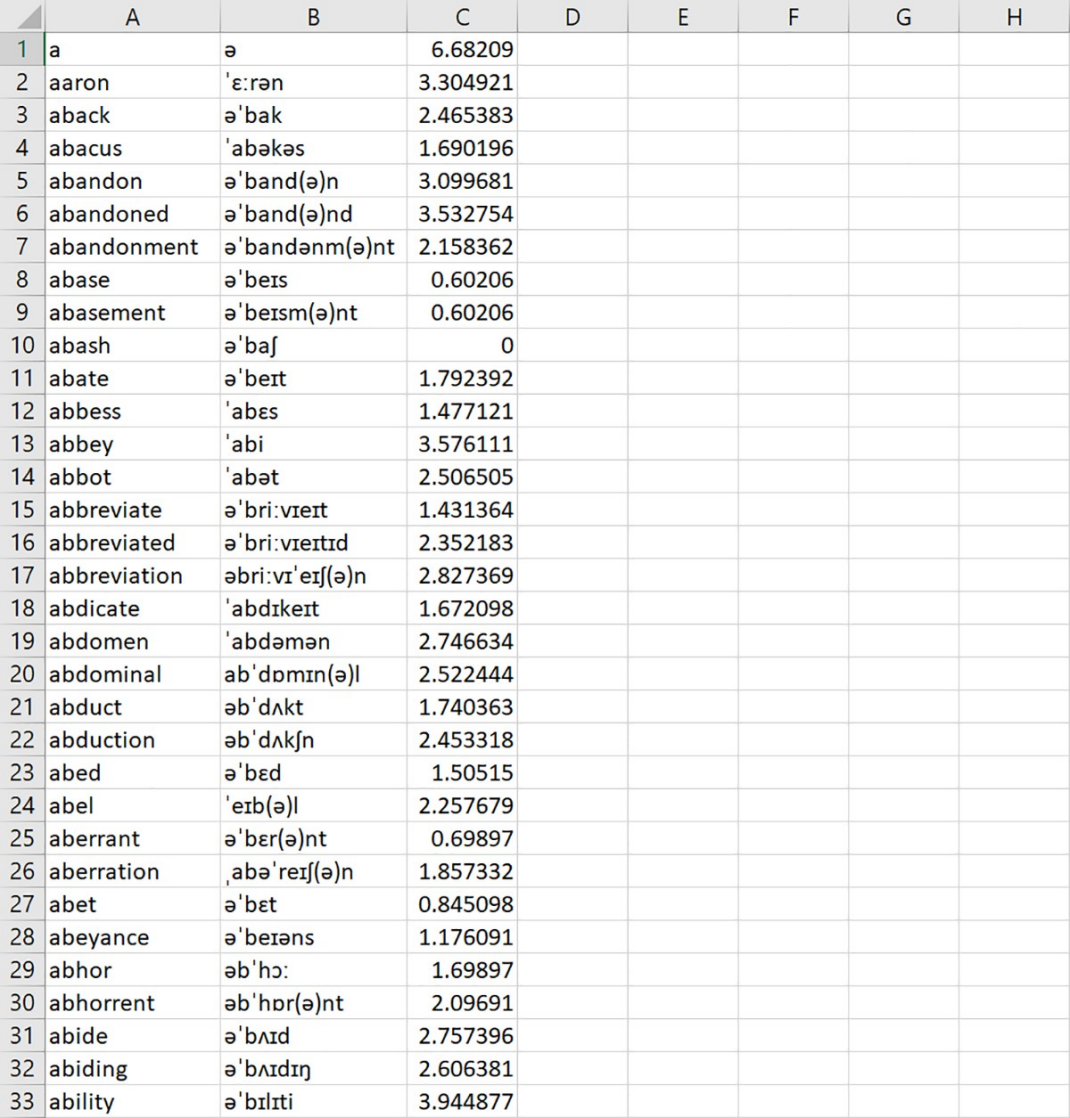
Example of a CSV UTF-8 (comma delimited) corpus file. Orthographic forms are listed in the first column, and corresponding pronunciations (if applicable) are listed in the adjacent column. Word frequencies are listed in the final column.

As with the input file, LexiCAL reads corpus data from a user-specified corpus file. Users should save corpus data in a single CSV UTF-8 (comma delimited) file on their machine, without any column headers, and direct LexiCAL to the corpus file by using the ‘Browse’ option. Orthographic forms should be listed in the first column, and corresponding pronunciations, if applicable, should be listed in the adjacent column. Word frequencies should be listed in the final column. The preview window in LexiCAL will show a preview of the first 20 items in the corpus file once it has been selected. Fig 3 shows an example of the corpus file.

#### Phonetic system

LexiCAL recognizes pre-defined phonologies for English (US), English (UK), French, Spanish, Dutch, and German in two phonetic systems: International Phonetic Alphabet (IPA) and Speech Assessment Methods Phonetic Alphabet (SAMPA [21]). Additionally, for English (US), LexiCAL also recognizes Klattese symbols [10]. The IPA consists of a standardized series of phonetic symbols designed to represent all the sounds in the world’s languages, while SAMPA and Klattese are alternative computer-readable phonetic scripts using ASCII characters. Table 1 provides the list of phonetic symbols for each language that LexiCAL recognizes, for each phonetic system.

**Table 1:** List of phonetic symbols that LexiCAL recognizes for each language.

Language	Phonetic System	Type	Phonetic Symbols
English (US)	IPA	Consonants	b, d, dʒ, ð, f, ɡ, h, j, k, l, m, n, ŋ, p, r, s, ʃ, t, tʃ, θ, v, w, z, ʒ, ʍ, x, ʔ
		Vowels	ɪ, i, ɛ, æ, ɑ, ɔ, ʊ, u, ə, eɪ, aɪ, aʊ, oʊ, ɔɪ
	Klattese	Consonants	b, d, J, D, f, g, h, y, k, l, m, n, G, p, r, s, S, t, C, T, v, w, z, Z
		Vowels	I, i, E, @, a, c, U, u, ^, x, |, e, Y, W, o, O, R, X, N, M, L
	SAMPA	Consonants	b, d, dZ, D, f, g, h, j, k, l, m, n, N, p, r, s, S, t, tS, T, v, w, z, Z, W, x,?, 4
		Vowels	I, i, E, a, A, O, U, u, V, @, e, aI, aU, o, OI, 3`, @`
English (UK)	IPA	Consonants	b, d, dʒ, ð, f, ɡ, h, j, k, l, m, n, ŋ, p, r, s, ʃ, t, tʃ, θ, v, w, z, ʒ, ʍ, x, ʔ
		Vowels	ɪ, iː, i, ɛ, a, ɑː, ɒ, ɔː, ʊ, uː, ʌ, ə, əː, ɪə, ɛː, ʊə, eɪ, aʊ, ʌɪ, əʊ, ɔɪ
	SAMPA	Consonants	b, d, dZ, D, f, g, h, j, k, l, m, n, N, p, r, s, S, t, tS, T, v, w, z, Z, W, x,?
		Vowels	I, i:, i, E, a, A:, Q, O:, U, u:, V, @, @:, I@, E:, U@, eI, aU, VI, @U, OI
French	IPA	Consonants	b, d, f, ɡ, k, l, m, n, ɲ, ŋ, p, ʀ, s, ʃ, t, v, z, ʒ, j, w, ɥ
		Vowels	a, ɑ, e, ɛ, ɛː, ə, i, œ, ø, o, ɔ, u, y, ɑ̃, ɛ̃, œ̃, ɔ̃
	SAMPA	Consonants	b, d, f, g, k, l, m, n, J, N, p, R, s, S, t, v, z, Z, j, w, H
		Vowels	a, A, e, E, E:, @, i, 9, 2, o, O, u, y, a~, e~, 9~, o~
Spanish	IPA	Consonants	b, β, d, ð, f, ɡ, ɣ, ʝ, k, l, ʎ, m, n, ɲ, ŋ, p, r, ɾ, s, θ, t, tʃ, v, x, z, ʃ, j, w
		Vowels	a, u, e, i, o
	SAMPA	Consonants	b, B, d, D, f, g, G, jj, k, l, L, m, n, J, N, p, rr, r, s, T, t, tS, v, x, z, S, j, w
		Vowels	a, u, e, i, o
Dutch	IPA	Consonants	b, d, f, ɣ, h, j, k, l, m, n, ŋ, p, r, s, t, v, ʋ, x, z, ɡ, c, ɲ, ʃ, ʒ
		Vowels	ɑ, ɛ, ɪ, ɔ, ʏ, ə, aː, eː, i, oː, y, øː, u, ɛi, œy, ɑu, ɑi, ɔi, iu, yu, ui, aːi, eːu, oːi, iː, yː, uː, ɔː, ɛː, œː, ɑː, ɑ̃, ɛ̃, ɔ̃, œ̃
	SAMPA	Consonants	b, d, f, G, h, j, k, l, m, n, N, p, r, s, t, v, P, x, z, g, c, J, S, Z
		Vowels	A, E, I, O, Y, @, a:, e:, i, o:, y, 2:, u, Ei, 9y, Au, Ai, Oi, iu, yu, ui, a:i, e:u, o:i, i:, y:, u:, O:, E:, 9:, A:, A~, E~:, O~, 9~
German	IPA	Consonants	b, ç, d, f, ɡ, h, j, k, l, m, n, ŋ, p, pf, r, s, ʃ, t, ts, v, x, z, ʔ, tʃ, dʒ, ʒ, i̯, u̯
		Vowels	a, aː, ɛ, ɛː, eː, ɪ, iː, ɔ, oː, œ, øː, ʊ, uː, ʏ, yː, ə, aɪ, aʊ, ɔʏ, uɪ, ɐ, l̩, m̩, n̩
	SAMPA	Consonants	b, C, d, f, g, h, j, k, l, m, n, N, p, pf, R, s, S, t, ts, v, x, z,?, tS, dZ, Z
		Vowels	a, a:, E, E:, e:, I, i:, O, o:, 9, 2:, U, u:, Y, y:, @, aI, aU, OY, uI, 6

*Note*. Diphthongs (combinations of two vowels) are recognized as a single phoneme.

*Individual symbols are separated by a single comma (‘*,*’)*. *Klattese symbols are only available for English (US)*.

**Fig 4:**
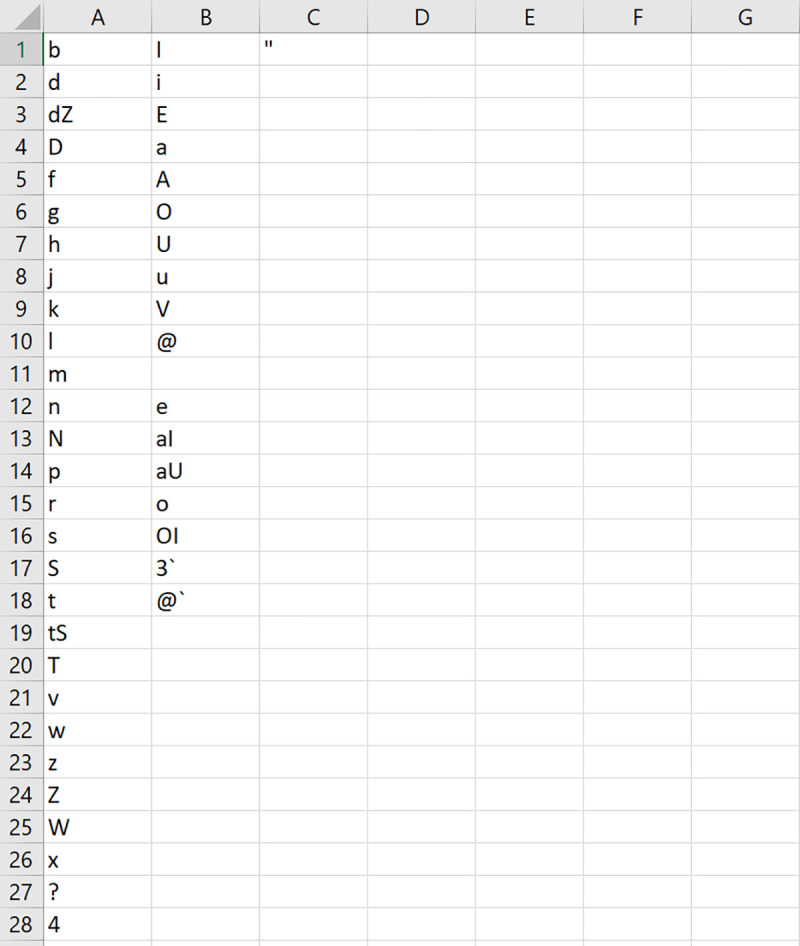
Example of a CSV UTF-8 (comma delimited) phonetic system file. Consonants are listed in the first column, and vowels are listed in the second column. The application will recognize diphthongs (combinations of two vowels) if they are listed as a single entity. The stress marking for primary stress is listed in the third column.

Other than the built-in phonetic systems, users can also choose to specify their own phonetic system by listing the phonetic symbols in a single CSV UTF-8 (comma delimited) file on their machine, without any column headers, and directing LexiCAL to the file by using the ‘Browse’ option. Phonetic symbols for consonants should be listed in the first column, and vowels in the second column. LexiCAL will recognize diphthongs (combinations of two vowels) as a single vowel if vowel combinations are listed as a single entity. The stress mark for primary stress should be listed in the third column. Fig 4 shows an example of a user-specified phonetic system file.

### Description of metrics

A list of the surface, orthographic, phonological, and phonographic metrics that are available in LexiCAL, including their descriptions and the algorithms used for calculations, are provided in Table 2.

**Table 2:** Description of the type of metrics available in LexiCAL.

Type of Metric	Description
Surface	
Stress code	Returns the stress pattern of the target word, based on which syllable the primary stress falls on.
Stress typicality	Returns the proportion of words in the corpus that share the same primary stress assignment as the target word, amongst all the words that have the same number of syllables as the target word [22].
Orthographic	
Length (No. of letters)	Returns the number of letters in the target word.
Neighbourhood density	Returns the number and the identity of orthographic neighbours the target word has in the corpus, based on single letter substitution (‘substitutions only’ option), or single letter substitution, addition, or deletion (‘substitutions, additions, and deletions’ option) [23,24].
Neighbourhood frequency	Returns the mean word frequency and standard deviation of the target word’s orthographic neighbours, based on single letter substitution (‘substitutions only’ option), or single letter substitution, addition, or deletion (‘substitutions, additions, and deletions’ option).
Orthographic Levenshtein Distance-20	Returns the mean Levenshtein edit distance and standard deviation of the target word’s 20 closest orthographic neighbours, based on single letter substitution, addition, or deletion [25].
Orthographic spread	Returns the number of letters in the target word that can be substituted to form an orthographic neighbour [26].
Uniqueness point	Returns the point where the target word orthographically diverges from all other words in the corpus, measured in terms of number of letters starting from the first letter [27].
Clustering coefficient (C coefficient)	Returns the proportion of orthographic neighbours of a target word that are also orthographic neighbours of each other, based on single letter substitution, addition, or deletion [28].
Sum bigram frequency	Returns the sum of the log-transformed frequencies of bigrams in the target word. The calculation of each bigram frequency takes into account the letter positions where the bigram occurs, so each bigram frequency is position-specific.
Phonological	
No. of phonemes	Returns the number of phonemes in the target word.
No. of syllables	Returns the number of syllables in the target words, based on the number of vowels.
Neighbourhood density	Returns the number and the identity of phonological neighbours the target word has in the corpus, based on single phoneme substitution (‘substitutions only’ option), or single phoneme substitution, addition, or deletion (‘substitutions, additions, and deletions’ option) [23,24].
Neighbourhood frequency	Returns the mean word frequency and standard deviation of the target word’s phonological neighbours, based on single phoneme substitution (‘substitutions only’ option), or single phoneme substitution, addition, or deletion (‘substitutions, additions, and deletions’ option).
Phonological Levenshtein Distance-20	Returns the mean Levenshtein edit distance and standard deviation of the target word’s 20 closest phonological neighbours, based on single phoneme substitution, addition, or deletion [25].
Phonological spread	Returns the number of phonemes in the target word that can be substituted to form a phonological neighbour [29].
Uniqueness point	Returns the point where the target word phonologically diverges from all other words in the corpus, measured in terms of number of phonemes starting from the first phoneme [30].
Clustering coefficient (C coefficient)	Returns the proportion of phonological neighbours of a target word that are also phonological neighbours of each other, based on single phoneme substitution, addition, or deletion [28].
Sum biphone frequency	Returns the sum of the log-transformed frequencies of biphones in the target word. The calculation of each biphone frequency takes into account the phoneme positions where the biphone occurs, so each biphone frequency is position-specific [10].
Phonographic	
Neighbourhood density	Returns the number and the identity of phonographic neighbours the target word has in the corpus, based on single letter and phoneme substitution (‘substitutions only’ option), or single letter and phoneme substitution, addition, or deletion (‘substitutions, additions, and deletions’ option) [31].
Neighbourhood frequency	Returns the mean word frequency and standard deviation of the target word’s phonographic neighbours, based on single letter and phoneme substitution (‘substitutions only’ option), or single letter and phoneme substitution, addition, or deletion (‘substitutions, additions, and deletions’ option).
Clustering coefficient (C coefficient)	Returns the proportion of phonographic neighbours of a target word that are also phonographic neighbours of each other, based on single letter and phoneme substitution, addition, or deletion.

#### Homophones and homographs

When computing any metric that involves a target word’s neighbours (i.e., neighbourhood density, neighbourhood frequency, Levenshtein distance-20, spread, uniqueness point, and clustering coefficient) LexiCAL will exclude the target word’s homographs (for orthographic metrics) and/or homophones (for phonological metrics) in the corpus from calculation. By way of illustration, the phonological neighbourhood of the target word “pail” (IPA: /peɪl/) will not include the word “pale” (IPA: /peɪl/), because it is a homophone of the target word. Some databases, such as the ELP [1], provides users the option of including the target word’s homophones in the neighbourhood count, but LexiCAL treats instances of the same spelling and/or pronunciation as a single representation in the lexicon.

By extension, homographs and/or homophones amongst the target word’s neighbours are also counted only once. For example, if the phonological neighbourhood of the word “sail” (IPA: /seɪl/) includes both the words “mail” (IPA: /meɪl/) and “male” (IPA: /meɪl/), LexiCAL will treat “mail/male” as a single phonological neighbour.

For any computation of frequencies involving a target word’s neighbours (i.e., neighbourhood frequency), the word frequencies of homographs (for orthographic metrics) and/or homophones (for phonological metrics) in the target word’s neighbourhood will be summed to give the combined frequency for that particular orthographic and/or phonological neighbour. With reference to earlier examples, the word frequency for “mail/male”, as a phonological neighbour of “sail”, will be the summed frequency of “mail” and “male”.

### Using LexiCAL

Once the input file, corpus file, and phonetic system have been selected, users should specify whether their input and corpus files contain only orthographic forms, or both orthographic forms and pronunciations. If the input and corpus files contain only orthographic forms, users will not be allowed to select surface, phonological, and phonographic metrics for calculation. If no stress mark has been specified in the phonetic system, surface metrics would not be returned.

Users can then select variables to be calculated by checking the respective options on the interface. The ‘Default’ button will automatically select number of phonemes, number of syllables, orthographic and phonological neighbourhood densities and frequencies (substitutions, additions, and deletions), and orthographic and phonological Levenshtein distance-20 to be calculated. The ‘Clear’ button will clear all selections.

Users can then press ‘Run’ to begin calculating the metrics, which will prompt users to specify the output file name and location on the directory for the output file to be saved. Progress will be reflected on the progress bar, and the output data will be saved as a single CSV UTF-8 (comma delimited) file. The column headings for each metric in the output file is listed in Table 3. If the application cannot run, an error message will be shown. A list of error messages and their solutions are listed in Table 4. To cancel any calculations already in progress, users can press the ‘Abort’ button.

**Table 3:** Column headings for each metric in the output file.

Type of Metric	Column Heading(s)
Surface	
Stress code	Stress Code
Stress typicality	Stress Typicality
Orthographic	
Length (No. of letters)	Length
Neighbourhood density	Orthographic Neighbourhood Density, Identity of Orthographic neighbours
Neighbourhood frequency	Orthographic Neighbourhood Frequency (M), Orthographic Neighbourhood Frequency (SD)
Orthographic Levenshtein Distance-20	OLD-20 (M), OLD-20 (SD)
Orthographic spread	Orthographic Spread
Uniqueness point	Orthographic Uniqueness Point
C coefficient	Orthographic C Coefficient
Sum bigram frequency	Sum Bigram Frequency
Phonological	
No. of phonemes	No. of Phonemes
No. of syllables	No. of Syllables
Neighbourhood density	Phonological Neighbourhood Density, Identity of Phonological neighbours (O), Identity of Phonological neighbours (P)
Neighbourhood frequency	Phonological Neighbourhood Frequency (M), Phonological Neighbourhood Frequency (SD)
Phonological Levenshtein Distance-20	PLD-20 (M), PLD-20 (SD)
Phonological spread	Phonological Spread
Uniqueness point	Phonological Uniqueness Point
C coefficient	Phonological C Coefficient
Sum biphone frequency	Sum Biphone Frequency
Phonographic	
Neighbourhood density	Phonographic Neighbourhood Density, Identity of Phonographic neighbours (O), Identity of Phonographic neighbours (P)
Neighbourhood frequency	Phonographic Neighbourhood Frequency (M), Phonographic Neighbourhood Frequency (SD)
C coefficient	Phonographic C Coefficient

*Note*. M = mean, SD = standard deviation, O = orthographic forms, P = pronunciations.

*Individual column headings are separated by a single comma (‘*,*’)*.

**Table 4:** List of error messages in LexiCAL and their solutions.

Category	Error message	Description	Solution
Input file	"The specified input file cannot be found in the directory."	The specified input file no longer exists in the specified location.	Do not remove the file from the directory. Replace the file, or use “Browse” to select a new input file.
	"Unable to read input file. Please close the file if it is open."	The input file cannot be read, possibly because it is open.	Close the file, and make sure there is no other program accessing the file.
	"No input file is selected. Please select an input file."	No input file has been specified.	Select an input file on your machine using the “Browse” option.
	"There are empty cells in the input. Please check the input file."	There are empty cells in the input file.	Make sure that there are no empty cells in the file.
	"Unable to read input file. Please check that it is saved in CSV format."	The input file is saved in wrong format.	Ensure that the file is saved in the CSV UTF-8 (comma delimited) file format.
Corpus file	"The specified corpus file was not found in the directory."	The specified corpus file no longer exists in the specified location.	Do not remove the file from the directory. Replace the file, or use “Browse” to select a new corpus file.
	"Unable to read corpus file. Please close the file if it is open."	The corpus file cannot be read, possibly because it is open.	Close the file, and make sure there is no other program accessing the file.
	"No corpus file is selected. Please select a corpus file."	No corpus file has been specified.	Select a corpus file on your machine using the “Browse” option.
	"There are empty cells in the corpus. Please check the corpus file."	There are empty cells in the corpus file.	Make sure that there are no empty cells in the file.
	"Unable to read corpus file. Please check that it is saved in CSV format."	The corpus file is saved in the wrong format.	Ensure that the file is saved in the CSV UTF-8 (comma delimited) file format.
	"The corpus file format is incorrect. Please ensure that there are 3 columns."	The corpus file does not have orthographic forms, phonologies, and word frequencies specified.	Ensure that the data in the corpus file follows the format required.
	"Please ensure that only numbers are in the frequency column of the corpus file."	The word frequency column has non-decimal numbers.	Ensure that the word frequency column contains only numbers (0–9) and decimals (‘.’).
Phonetic system	"Unable to read phonetic system file. Please check that it is saved in CSV format."	The phonetic system file is saved in the wrong format.	Ensure that the file is saved in the CSV UTF-8 (comma delimited) file format.
	"The phonetic system file format is incorrect."	The phonetic system file does not have consonants, vowels, and stress marks specified.	Ensure that the data in the phonetic system file follows the format required.
	"The specified phonetic system file was not found in the directory."	The specified phonetic system file no longer exists in the specified location.	Do not remove the file from the directory. Replace the file, or use “Browse” to select a new phonetic system file.
	"No phonetic system file is selected. Please select a phonetic system file."	No phonetic system file has been specified.	Select a phonetic system file on your machine using the “Browse” option, or select one of the inbuilt phonetic systems.
	"Unable to read phonetic system file. Please close the file if it is open."	The phonetic system file cannot be read, possibly because it is open.	Close the file, and make sure there is no other program accessing the file.
Metrics	“No metrics have been selected.”	No metrics have been selected.	Select at least one metric.
	"Words (orthographic) must not be empty for orthographic metrics."	Orthographic metrics have been selected but the input file has no words in the first column.	Ensure that there are words in the second column.
	“Words (phonological) must not be empty for phonological metrics."	Phonological metrics have been selected but the input file has no pronunciations in the second column.	Ensure that there are pronunciations in the second column.
	"Words (phonological) and Words (orthographic) must not be empty for phonographic metrics."	Phonographic metrics have been selected but the input file has either no words in the first column or no pronunciations in the second column.	Ensure that there are words in the first column and pronunciations in the second column.
	"Words (phonological) must not be empty for surface metrics."	Surface metrics have been selected but the input file has no pronunciations in the second column.	Ensure that there are pronunciations in the second column.
	"Please specify a stress mark in the phonetic system."	Surface metrics have been selected but the phonetic system file has no stress marks in the third column.	Specify a stress mark in the third column of the phonetic system file.
Others	"Unable to tokenise the string “xxx”.	There is a character in the input or corpus file that LexiCAL does not recognize.	Ensure that you have selected the correct phonetic system, and that the pronunciations in your input or corpus file follow the list of symbols recognized.
	“The file [output filename.csv] is open. Please close it.	LexiCAL is unable to write to output file as it is open.	Close the output file. Do not open the output before operations are complete.
	"The corpus needs at least 20 unique items if OLD-20/PLD-20 is selected."	The number of items in the corpus file is less than 20, but PLD-20/OLD-20 has been selected.	Ensure that there are at least 20 unique items in the corpus file.

At any one time, only one instance of the LexiCAL can be open. This prevents users from running multiple instances of the program concurrently.

### Python scripts

In addition to the program, each download also comes with a set of 23 Python scripts that were used to compile the executable. The Python scripts are organized by each metric and function, and are well-documented along with comments on code and algorithmic descriptions. Users who are familiar with Python can therefore also choose to calculate the metrics by running individual Python modules on any operating system or platform that supports Python.

Users can also reproduce and/or modify these Python scripts to develop new metrics, or transform existing ones (e.g., adding a logarithmic transformation). The Python scripts can also be combined with other Python libraries to test new theories, or develop new research tools. Any redistribution and/or modification of the Python scripts, however, should be in accordance with the terms of the GNU General Public License as published by the Free Software Foundation, either version 3 of the License, or any later version (see further licensing information at the end of this article). Anyone reproducing any part of the source code, with or without modification, should also acknowledge and cite this article.

## Data validation

**Fig 5:**
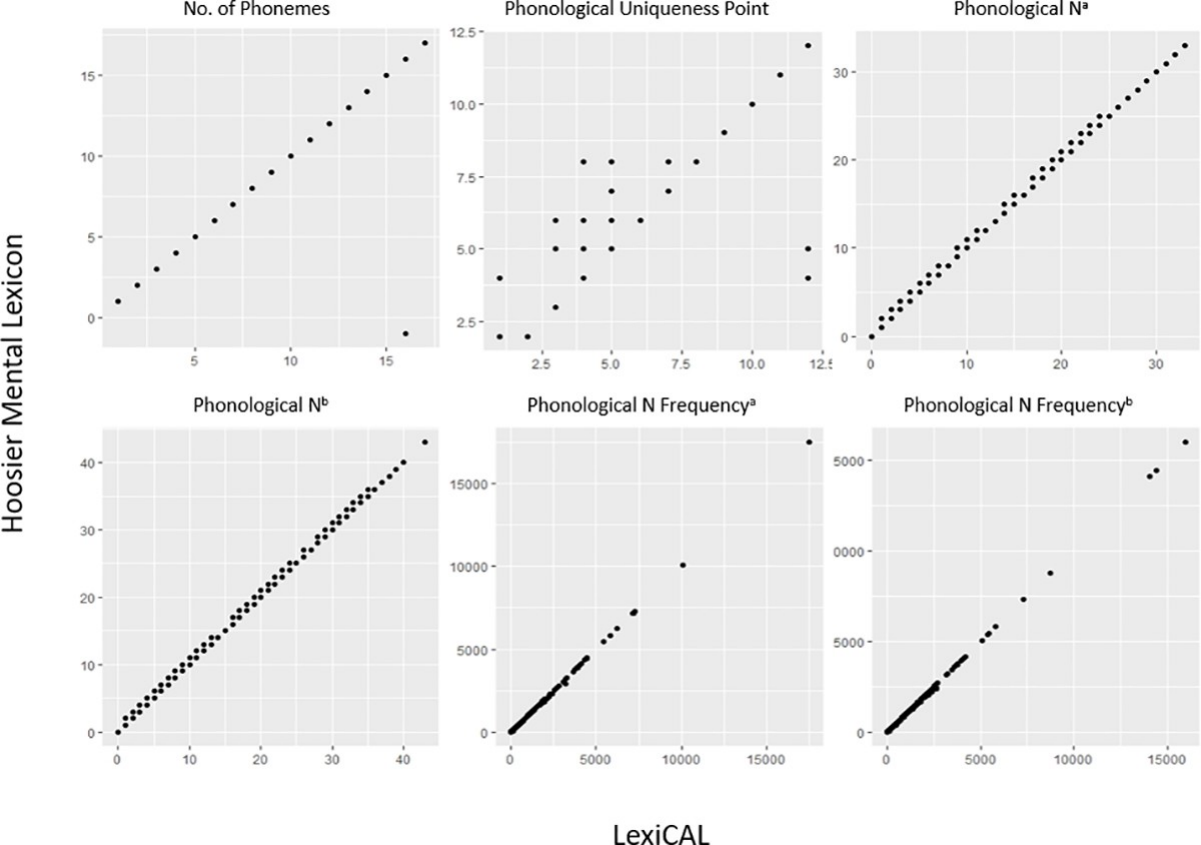
Scatterplots displaying the relationships between the data computed by LexiCAL, and the data from the Hoosier Mental Lexicon. ^a^ substitutions only; ^b^ substitutions, additions, and deletions.

**Fig 6:**
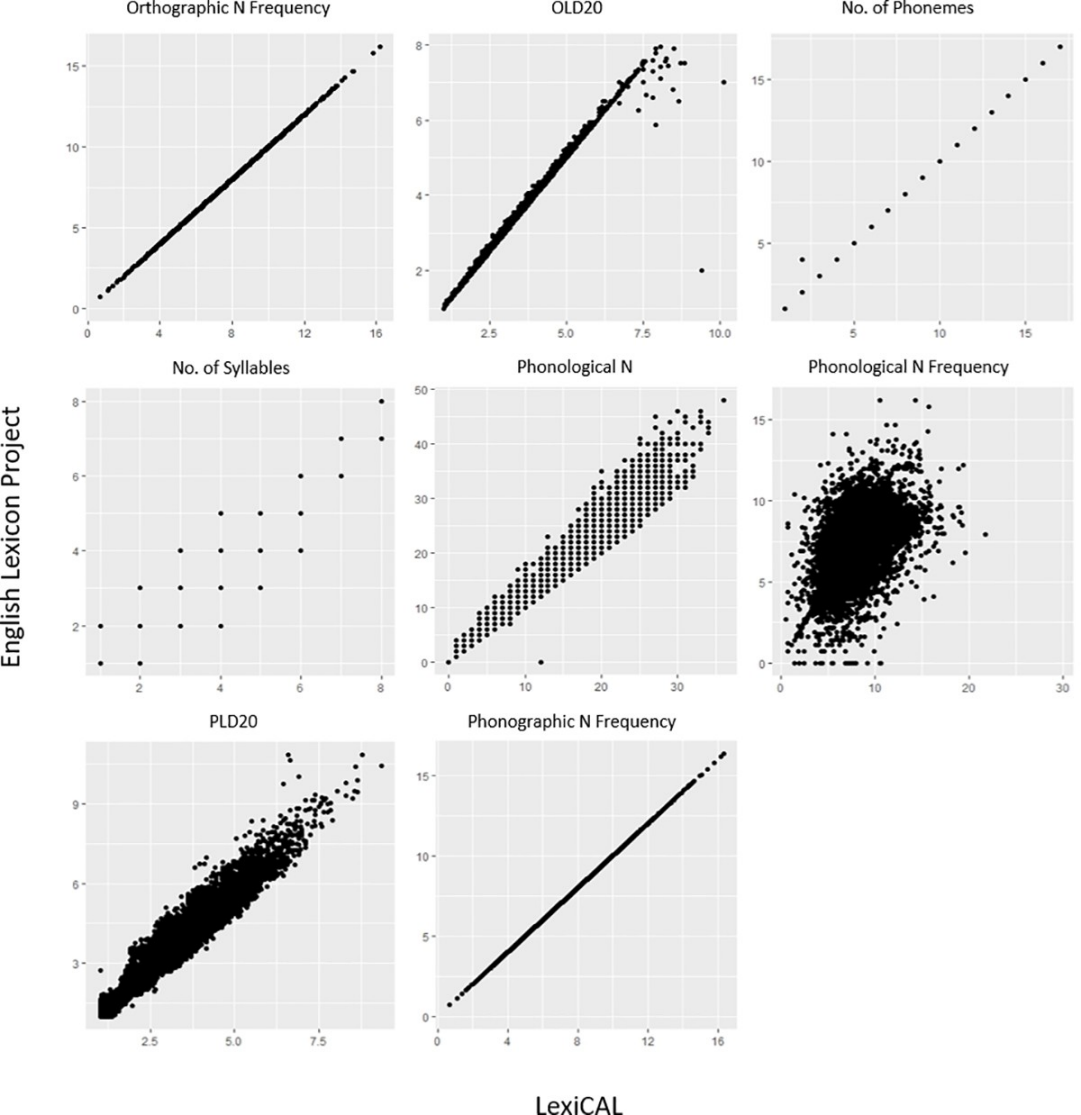
Scatterplots displaying the relationships between the data computed by LexiCAL, and the data from the English Lexicon Project. Scatterplots for word length, orthographic neighbourhood density, and phonographic neighbourhood density are not presented because the data were identical.

In order to establish the validity of the data calculated by LexiCAL, various lexical properties for the words in the HML (*n* = 19,321) [3] and the restricted ELP (*n* = 40,481) [1] were computed and then compared with the data provided by these databases. Table 5 presents the correlation coefficients and between the data computed by LexiCAL, and the data from the HML and the restricted ELP, while the scatterplots are presented in Figs 5 and 6. Despite the slight differences in the algorithms used (with respect to the treatment of homographs and homophones in the corpus), the scatterplots and correlations indicate that LexiCAL’s computations produce values that correspond very closely with these databases.

**Table 5:** Pearson correlation coefficients for the metrics computed by LexiCAL, and the data in the Hoosier Mental Lexicon (HML) and the restricted English Lexicon Project (ELP).

Metric	HML (*n* = 19,321)	ELP (*n* = 40,481)
Length (number of letters)	NA	1.00***
Orthographic neighbourhood density^a^	NA	1.00***
Orthographic neighbourhood frequency^a^	NA	1.00***
Orthographic Levenshtein distance-20 (OLD-20)	NA	.999***
Number of phonemes	.999***	1.00***
Number of syllables	NA	.983***
Phonological neighbourhood density^a^	1.00***	.990***
Phonological neighbourhood density^b^	1.00***	NA
Phonological neighbourhood frequency^a^	1.00***	.990***
Phonological neighbourhood frequency^b^	1.00***	NA
Phonological Levenshtein distance-20 (PLD-20)	NA	.970***
Phonological uniqueness point	.997***	NA
Phonographic neighbourhood density^a^	NA	1.00***
Phonographic neighbourhood frequency^a^	NA	1.00***

^a^ substitutions only.

^b^ substitutions, additions, and deletions.

*** *p <* .001.

## Conclusion

LexiCAL is a simple and intuitive application that improves on existing resources by offering researchers the flexibility of computing lexical variables for any stimuli with respect to any corpus of text, using any phonetic system (if applicable). Notably, the lexical variables that LexiCAL can compute include a broad range of inbuilt surface, orthographic, phonological, and phonographic metrics. From a methodological perspective, the program is a useful tool for researchers interested in calculating the lexical properties for any stimuli (both words and nonwords) with reference to any corpus, and should hopefully serve as a resource for experimental design, data analyses, and/or database creation.

## Licensing information

LexiCAL is a free software that can be redistributed and/or modified under the terms of the GNU General Public License as published by the Free Software Foundation, either version 3 of the License, or any later version. LexiCAL is distributed in the hope that it will be useful, but *without any warranty*; without even the implied warranty of merchantability or fitness for a particular purpose. See the GNU General Public License for more details.

LexiCAL makes use of open-source components. The source code for the open-source projects, along with their license information, is provided in the supplementary material.

## Supporting information

S1 File The program (LexiCAL.exe), along with the Python scripts used to compile the executable. (ZIP)
